# Monitoring Pharmacological Treatment in Patients With Chronic Noncancer Pain

**DOI:** 10.7759/cureus.20358

**Published:** 2021-12-12

**Authors:** Grisell Vargas-Schaffer, Allen Steverman, Veronique Potvin

**Affiliations:** 1 Anesthesiology and Pain Medicine, Pain Center Centre Hospitalier de l'Université de Montreal (CHUM), Montreal, CAN; 2 Family Medicine, Pain Center Centre Hospitalier de l'Université de Montreal (CHUM), Montreal, CAN; 3 Anesthesiology, Pain Center Centre Hospitalier de l'Université de Montreal (CHUM), Montreal, CAN

**Keywords:** chronic pain management, side effects of medical treatment, multimodal analesia, opioids use, opioids in chronic non cancer pain, chronic treatment, chronic non cancer pain

## Abstract

Chronic pain has been not recognized as a chronic illness, and its far-reaching impacts are often ignored. Chronic noncancer pain (CNCP) is a chronic disease and health care professionals need recommendations on how to monitor treatments, patients and long-term side effects of the different medications used to control CNCP.

CNCP patients make up a vulnerable population due to the various associated pathologies and the challenging socio-economic conditions experienced by many of these patients. CNCP is more common among older adults, females, cancer survivors, indigenous peoples, veterans, and populations affected by social inequities and discrimination. These social determinants can lead to a complex interplay between chronic pain, mental illness, and substance use disorders. Given these realities, long-term pharmacological and side effect surveillance is more complex.

Follow-up of patients with CNCP is a challenge for physicians, and thus it is important to provide recommendations on how to monitor treatments and long-term side effects of the different medications used to control CNCP.

## Introduction and background

Pain continues to be one of the main reasons for medical consultation worldwide. Chronic noncancer pain (CNCP) is a common condition considered to be an epidemic with major social and economic impacts [1-4]. According to The International Association for the Study of Pain’s Declaration of Montreal, access to pain management is a fundamental human right [5]. Adequate pain management should be a priority in medical care, with proper assessment and appropriate treatments, including pharmacological and nonpharmacological approaches.

Multimodal analgesia includes the use of two or more drugs that act via different mechanisms to provide analgesia. The aim of multimodal analgesia is to reduce opioid requirements, improve pain relief, and decrease side effects. As physicians move away from an opioid-centric model in pain management, the focus has shifted to multimodal analgesia.

Considering its multifactorial etiology, the treatment of CNCP requires a comprehensive, integrated, and multifaceted model of care. Most treatment guidelines recommend multimodal therapy [6-8]. The prescription of analgesic medication requires careful attention to both the dosing schedule and monitoring side effects. Despite controversies surrounding pharmacological treatments in CNCP, rational and appropriate use of opioids continue to be one of the valid tools within a multimodal comprehensive treatment plan. Although the use of drug combinations can provide the benefit of pharmacological synergism, it also increases the risk of potential side effects and the need for long-term monitoring. Long-term treatment regimens with multimodal treatment, even at lower doses, require long-term surveillance.

The objective of this update was to gather appropriate monitoring parameters for the long-term management of patients suffering from CNCP and provide family physicians with a practical and comprehensive overview to help guide follow-up.

Quality of evidence

This article is a literature review and presents an approach for monitoring the potential side effects of medications used in the long-term treatment of patients with CNCP. The relevance of the article was identified after an internal audit of 85 medical charts at a tertiary care center pain clinic (Montreal University Hospital Center [CHUM]) revealed that only 16% of the patients had received adequate screening for hypogonadism. This result led us to consider the likelihood that, if physicians working in a tertiary care university center are not carrying out adequate monitoring for long-term side effects of pharmacological treatments used to control CNCP, other medical specialists and family doctors are probably not carrying this out either.

Unfortunately, at this time there is a lack of evidence-based recommendations for the monitoring of patients being treated for CNCP. This article presents a concise summary of the essential elements that should be monitored for all patients receiving treatment for CNCP to help palliate this lack of guidelines.

## Review

Long-term treatment regimens that combine co-analgesics such as antidepressants and anticonvulsants with opioids, even at lower doses, require long-term surveillance. Indeed, this patient who receives small doses of three or more drugs must be monitored for possible alterations in cognitive function, mood, concentration, gastrointestinal function, hypothalamic-pituitary-adrenal-gonadal axis (HPAG axis), sexual function, and osteoporosis.

**Figure 1:**
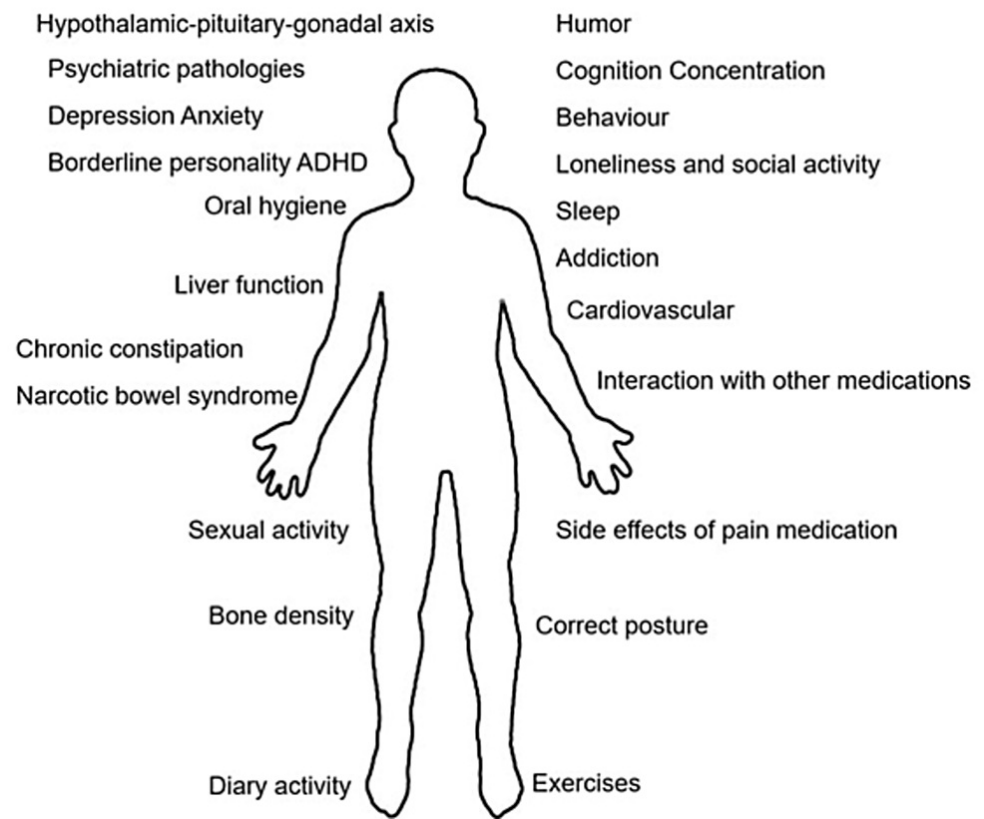
Monitoring treatment in patients with chronic noncancer pain

Taking into consideration that multimodal analgesia is the most used for the treatment of CNCP, we propose that the most important factors to monitor from the pharmacological point of view in the long-term follow-up of patients treated for CNCP are the following: oral hygiene, chronic constipation, and narcotic bowel syndrome (NBS), cardiovascular opioids effects, effects on the immune system, sleep and somnolence, respiratory depression, substance use disorders, and addiction, HPA axis and hypothalamic-pituitary-gonadal axis (HPGA) axis dysregulation, mental health and lack of exercise (Figure 1).

Oral hygiene

Most medications used to treat pain decrease saliva production and the oral mucus can, therefore, be very dry. Physiologic amounts of salivary secretions are essential for oral health. Saliva influences various processes in the oral cavity such as protection from cavities, as well as digestive and immunologic functions [9].

Opioid use increases the risk for a variety of oral health conditions, including dental caries and periodontitis. Furthermore, opioids have been shown to directly induce heightened cravings for sweet carbohydrates [10]. It is imperative to educate patients on the need to increase oral hygiene when receiving pharmacological treatments for CNCP.

Chronic constipation and NBS

The multimodal approach also introduces multiple potential sources for constipation since many drugs used as coanalgesics can cause constipation. Chronic constipation is defined as having two or more of the following symptoms in the last 12 months: lumpy or hard stools, fewer than three defecations per week, the sensation of incomplete evacuation, and/or anorectal obstruction/blockage and need for manual maneuvers to facilitate defecation [11].

NBS is characterized by chronic or frequently occurring abdominal pain that worsens with continued or escalating doses of opioids [11]. This pain should not be associated with a new cause of pain or another associated pathology. NBS was defined as the presence of severe abdominal pain daily for more than three months duration when taking more than 100 mg of morphine equivalent per day [11]. Opioid bowel dysfunction (OBD) is defined as the presence of reflux, nausea and vomiting, severe constipation, and abdominal distension [11].

In addition, the lack of regular physical activity often observed in patients with CNCP is another factor that can contribute to constipation.

Cardiovascular opioid effects

Although opioids have been used in the treatment of CNCP for decades, only a few studies have looked specifically at the relationship between opioid use and cardiovascular effects [12]. Several studies [13-15] have found an increased cardiovascular risk, up to 77%, in patients who receive opioids for the treatment of CNCP (Table 1) [12-16].

**Table 1:** Cardiac effects of chronic opioid therapy There are limited data to suggest that chronic opioid administration may be associated with an increased risk for cardiac-related adverse effects. However, this observation has not yet been confirmed. All opioids associated with other opioids, benzodiazepines, antidepressants, anticonvulsants and muscle relaxants, can lead to serious side effects including respiratory distress and bradycardia.

Opioid	Cardiovascular effects	Medication Interaction - cardiac effect
Buprenorphine	Not thought to have any direct negative effects on cardiac function. However, buprenorphine administration can lead to hypotension, orthostatic hypotension and syncope. May have a dose related effect on QTc.	Patients taking Buprenorphine with quinidine, procainamide, disopyramide, sotalol, amiodarone, and dofetilide may also be at increased risk of prolonged QTc.
Tramadol	Low risk for cardiovascular adverse effects when used at recommended doses Cardiac side effects may range from agitation and palpitations to rhythm abnormalities, conduction defects, and cardiac arrest.	There is a risk for serotonin syndrome when associated with antidepressants, which can lead to cardiac arrhythmia.
Tapentadol	Low risk of cardiovascular adverse events at doses used for chronic analgesia.	There is a risk for serotonin syndrome when associated with antidepressants, which can lead to cardiac arrhythmia. There is a risk for hypotension when used in combination with other CNS depressants, or in patients with significantly compromised cardiac function.
Morphine	Morphine can cause histamine release resulting in vasodilation and hypotension.	Increased risk of cardiac impairment and myocardial infarction when combined with Clonidine.
Oxycodone	Oxycodone is not thought to have significant adverse effects on cardiac function. Oxycodone can cause bradycardia and hypotension, including orthostatic hypotension. Oxycodone has been reported to lead to a modest dose-related increase in QTc.	Persistent Bradycardia with the Long-term use of Phenytoin and Oxycodone. Oxycodone can cause bradycardia and hypotension when associated with histamine release like Quinine.
Hydromorphone	Hydromorphone can cause hypotension, including orthostatic hypotension and syncope related to histamine release.	Hydromorphone is related to various cardiac side effects such as histamine release, which leads to bradycardia, vasodilatation, and hypotension, decreasing cardiac output especially when used in combination with benzodiazepines.
Fentanyl	Minimal changes to cardiovascular function other than (usually) modest changes in heart rate and blood pressure. Fentanyl administration in analgesic doses can cause hypotension	Fentanyl combined with benzodiazepines can lead to profound cardiovascular changes, including decreased stroke volume and cardiac output, as well as profound decreases in blood pressure.
Methadone	Methadone can cause edema, as well as syncope, flushing and hypotension. Increased risk for QTc prolongation which can lead to torsade de point. Risk for QTc prolongation appears to increase with increased methadone dose. Methadone influences cardiac conductivity, can cause edema, as well as syncope, flushing and hypotension.	Although it is a relatively rare side effect. Methadone combined with Quetiapine can increase the risk of arrythmia that may be serious and potentially life-threatening. Caution required when using other medications that also increase the risk for QTc prolongation

Interaction with other medications

When treating patients with multiple comorbidities, the use of different medications increases the potential for drug interactions. Pharmacological treatment in CNCP, therefore, requires vigilance regarding potential drug interactions. Noting that this is not meant to be a comprehensive review of all possible interactions, some of the most frequent interactions seen in the follow-up of patients treated for CNCP are highlighted here.

Drug-drug interactions are well recognized and are classified into three types: pharmacokinetic, pharmacodynamics, and pharmaceutical. Certain antidepressants combined with opioids can increase the risk of serotonin syndrome [17]. Other medication interactions that require monitoring include between duloxetine, venlafaxine, citalopram, fluoxetine, phenelzine, and certain opioids such as fentanyl and methadone. Caution is also advised when combing fluvoxamine or citalopram with oxycodone.

It is important to note that methadone, meperidine, and fentanyl inhibit the reuptake of serotonin and their use with certain antidepressants can contribute to serotonin syndrome. Furthermore, methadone plasma concentrations can be increased by concurrent use of certain antimicrobials (fluconazole, ciprofloxacin, and clarithromycin), certain antidepressants (fluoxetine and amitriptyline) as well as certain antipsychotics such as quetiapine.

The most frequent interactions between medications commonly used for pain management and other medications involve opioids, SSRIs, benzodiazepines, phenothiazine, dexamethasone, imidazole, thyroxine, furosemide, and anticonvulsants.

Effects on the immune system

Given that many chronic pain patients tend to be elderly and present multiple comorbidities, potential immunosuppression in these patients needs to be considered.

It has been well documented that with chronic stress (and chronic pain as a variant) and chronic inflammation, the interaction of the nervous, endocrine, and immune systems can contribute to potential depressive symptoms. In addition, depression and the use of antidepressants can negatively impact the immune system [18,19].

Opioids directly affect the μ-opioid receptor on all immune cells. They may also modulate the immune function indirectly through glucocorticoids released by the hypothalamic-pituitary-adrenal (HPA) axis and norepinephrine released by the sympathetic nervous system.

Sleep and somnolence

A large majority of patients with CNCP suffer from insomnia and sleep disorders. In addition, several studies report an alarming relationship between sleep apnea and opioid use, as well as instances of death due to opioid overdose.

Orally administered opioid drugs, even at low doses, decrease time spent in the deep stages of sleep, with an accompanying increase in stage 2 sleep. The change in deep sleep is remarkable, as it represents a 30% to 50% decrease in stage 3 and stage 4 sleep [20].

There is evidence to support the benefits of antidepressants and anticonvulsants with improvements in pain scores, sleep, and quality of life. However, these benefits come at the expense of increased daytime somnolence as well as possible alterations in HPGA function with long-term use.

Respiratory depression 

Respiratory depression remains the most serious potential side effect of opioids with an ultimate risk of death. The mechanism of opioid-induced respiratory depression involves Mu opioid receptor inhibition of the brainstem respiratory control centers impacting both rate and depth of respiration, eventually resulting in increased arterial partial pressure of carbon dioxide and reduced partial pressure of oxygen [21]. This risk of respiratory depression is a major limiting factor for dose escalation in providing effective analgesia.

All opioids combined with benzodiazepines, antidepressants, anticonvulsants, and muscle relaxants can lead to serious side effects including respiratory distress.

HPA axis and HPGA

The HPA axis is involved in the neurobiology of mood disorders, including depression and bipolar disorder, posttraumatic stress disorder, anxiety disorder, ADHD, borderline personality disorder, and addictions including alcoholism. The HPA is implicated in other functional diseases like irritable bowel syndrome and chronic fatigue among others [22,23].

All opioids, endogenous and exogenous, modulate gonadal function primarily by acting on opioid receptors in the hypothalamus. Hypogonadism can lead to irregular menstrual cycles or amenorrhea in women, erectile dysfunction in men, infertility, and decreased libido in both sexes. Symptoms like flushing, sweating, lethargy, sleep disturbance, loss of body hearing, decreased muscle mass, osteopenia or osteoporosis, and depression are also common in both sexes. Hypogonadism secondary to opioids is more prevalent in men than in women and opioid-induced hypogonadism in CNCP is underrecognized and undertreated. According to some authors, the prevalence may be higher in patients receiving high doses of opioids, especially via the intrathecal route [22,23].

The HPA axis is also affected by many other medications used to control CNCP including antidepressants, and anticonvulsants [23] which are the main co-analgesic drugs used to manage CNCP.

Mental health

According to the World Health Organization (WHO), mental health is a “state of well-being in which the individual realizes his or her own abilities, can cope with the normal stresses of life, can work productively and fruitfully, and is able to contribute to his or her community” [24].

Co-morbidities that can accompany chronic pain include depression, anxiety, borderline personality disorder, and adult attention-deficit/hyperactivity disorder (ADHD) among others. CNCP pain is a significant risk factor for the development of a mental health disorder in the adult general population [22-27].

There are an increasing number of patients with chronic pain who present with symptoms of ADHD. Recent studies suggest that up to 80% of adults with ADHD have generalized pain, as compared to 17.4% in a control population [28].

Substance use disorders and addiction

The abuse of opioid prescriptions has been in the media spotlight in recent years. This attention has contributed to the stigmatization and social isolation of patients who require these drugs to help control their CNCP. While it remains imperative that we avoid being opiophobic or opiophilic, opioids can be used to treat chronic pain responsibly by providing adequate information to our patients [29-31]. There is a need to refine the way opioids are prescribed by appropriately selecting patients and prescribing opioids to “control physical pain” and not to relieve the psychosocial suffering that usually accompanies chronic pain [30,31].

The inappropriate use of opioids will always exist, and it is our duty to inform our patients of this risk and establish guidelines for use. Despite popular belief, the majority of patients followed in pain centers for CNCP are not at high risk of misuse/abuse, nor are they taking inappropriate amounts of opioids [29].

Lack of exercise

According to the WHO “Regular physical activity is proven to help prevent and treat noncommunicable diseases (NCDs) such as heart disease, stroke, diabetes and breast, and colon cancer. It also helps to prevent hypertension and obesity, and can improve mental health, quality of life and well-being.”

Numerous articles highlight exercise as an important part of long-term pain management. Current evidence suggests physical activity and exercise is an intervention with few adverse events that may improve physical function, decrease pain severity, and enhance the quality of life [32-35].

Finally, Table 2 highlights the key points to monitor in the long-term treatment of patients with CNCP.

**Table 2:** Checklist for monitoring treatment for chronic noncancer pain

Patients with chronic pain	What to do and watch for
Patients with CNCP between the ages of 18-55 receiving opioids and coanalgesics for more than one year.	Monitor hormones (cortisol, ACTH, TSH, T3, T4, testosterone) once a year.
Patients with CNCP over the age of 55 receiving opioids and coanalgesics for more than one year.	Monitor hormones once a year. Bone density scan every 2 years is recommended.
Patients with dry mouth	Monitor oral health.
Patients receiving opioids for more over 1 year with concurrent cardiac disease.	Monitoring cardiac function. ECG once a year.
Patients receiving pharmacological treatments for comorbid pathologies.	Monitor for drug interactions.
Patients receiving opioids can be at increased risk for respiratory depression.	Monitor for drug interactions and potentiation effects of different drugs such as benzodiazepines + opioids. Consider discussing a naloxone kit with the patient and their family.
All patients being treated for CNCP.	Frequently monitor emotional and mental health status.

## Conclusions

Chronic pain sufferers make up a vulnerable population of patients, often presenting multiple health challenges that must be monitored and treated accordingly to improve quality of life.

Within an integrative approach, rigorous surveillance of the medium and long-term side effects of the medications used to control the CNCP needs to be implemented.

We consider this article to be important, an opportunity to present and summarize the parameters to be monitored when treating a patient with CNCP. Unfortunately, to date,, there are no evidence-based recommendations to support monitoring strategies in patients treated for CNCP.

## Figures and Tables

**Figure 2:**
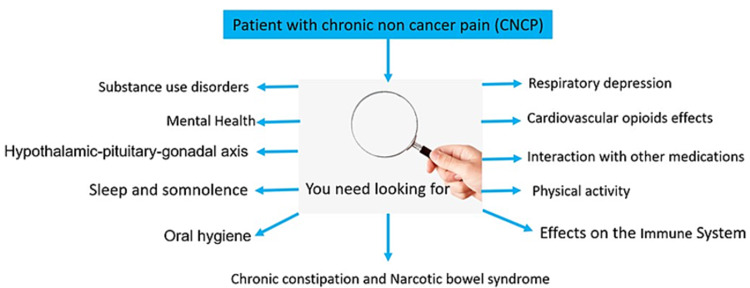
Graphical abstract
